# From *an Essay on Human Combustion from the Abuse of Spirituous Liquors*

**Published:** 1800-07

**Authors:** Pieree-Aimee Lair

**Affiliations:** Paris


From *An Eſſay on Human Combuſtion from the Abuſe of Spirituous Liquors*, by PIEREE-AIMEE LAIR, Paris, 1 Vol. duodecimo, we preſent our Readers with the following Extracts.

THE bodies of living perſons have been known to catch fire from the flame of other bodies, and continue to burn almoſt to aſhes.

On this aſtoniſhing and little known fact, the author ventures, as he ſays, to make ſome obſervations, which are the ſubſtance of his eſſay.

He begins by remarking, that the fact itſelf may be difficult to be believed, and will find the more opponents becauſe of its rarity; and becauſe there are examples of parts of the human body, to which this fatal accident has happened, that have not been conſumed when they have not been abſolutely ſubjected to the action of the fire. But he adds, this phenomenon has been aſſerted, as true, by too many learned men, of undoubted veracity, for it to be called in queſtion. The names of thoſe he cites are Bianchini, Maffei, Rolli, Lecat, and Vicq-d'Azyr.

He enters on his ſubject by obſerving, that he ſhall have recourſe to facts; which are much more ſatifactory than ſuppoſitions. Moſt of thoſe he cites, which are not a few, are extracted from the journals of various nations; and, with ſome ſhades of difference, offer much the ſame reſults, which are thus enumerated by the author:

1. The perſons who have been ſubject to combuſtion, have been long accuſtomed to drink ſpirituous liquors.

2. None but women have been conſumed.

3. Theſe women were old.

4. The body has not burned ſpontaneouſly, but accidentally.

5. The extremities of the body, ſuch as the feet and hands, have generally been ſpared by the fire.

6. Water

6. Water, inftead of extinguifhing the fire in thofe parts that were in a ftate of combuftion, has fometimes only given it greater activity.

7. The fire has little injured, and fometimes not at all, thofe combuftible things that were in contact with the body when it was burning.

8. The combuftion of thefe bodies has left a refidue of greafy and fetid afhes and fat, that were unctuous, offenfive to the fmell, and very penetrating.

It is proper to add, that often, while the fire was in the height of its action, it fent forth little or no flame.

Having eftablifhed this feries of facts, he examines each, individually, and begins with the abufe of fpirituous liquors, to which moft of the victims of this horrible death had been fubject. In order to fhew how this practice may render the body combuftible, he remarks, it is generally known, that people who drink much eat little; which little is of high-feafoned viands; that their urine is aqueous, and that the alcoholic parts, like all volatile fubftances, are but little decompofed, penetrate the mufcles, and as it were, faturate the whole mafs. In proof of this he cites different cafes; of which it has been remarked, that the bodies of perfons who have died drunk, fend forth a vinous odour long after death. But, without fuppofing fo much, there are few who have not been fenfible of the alcoholic vapours which drunkards exhale. Two confequences refult from a body being thus impregnated with alcohol; one is, that the vinous fpirit dries the flefh, foftens the fat, and fits them both for combuftion; and the other, that this kind of internal and highly inflammable vapour-bath is ever ready to catch fire, and to communicate it to the body that is already thus prepared.

Combuftion has only been obferved to take place in women; which does not prove that men are wholly exempt from it, but that they are lefs fubject to it than the other fex. This propenfity the author attributes to the delicacy of the female fibres, and the lefs compactnefs of the parts of the body, which is natural to them, and their fedentary life, rendering both the flefh and the fat more proper to abforb fpirituous fubftances, and difpofe them to inflammation.

The women were old: the love of liquors is the fucceffor of the paffions of youth, and is moft manifeft, particularly in women, in an advanced age. The little exercife of which this feafon of life admits, encourages the increafe of inflammable fubftances in the body, and thus gives place to the accident in queftion.

Was the fire fpontaneous? or was it communicated by an ignited ignited body? The latter cafe is admitted by the author in fupport of his opinion. He obferves, it is poffible that thefe inflammable vapours, which emanate from the bodies of drunken people, may catch fire, and communicate it to the bodies by which they are exhaled. Perfons are cited who had drunk brandy, and whofe breath, kindled by the flame of a candle, had communicated the fire inwardly. In confirmation that the fire had caught by accident in all the cafes he quotes, it was obferved, that the remains of moft of the perfons were found near the fire.

The author then returns to the poffibility of fpontaneous combuftion in the human frame; admitted by fome enlightened obfervers. Having noticed, that thefe kind of conflagrations take place in the mineral and vegetable kingdoms, he cites cafes of men, after drinking much brandy, that have breathed flames: but fuppofing thefe facts confirmed, he does not imagine fuch inflammation could totally deftroy the body. To this it may be replied, according to the author's own doctrine, that fince he fuppofes bodies may take fire from a pipe, a lighted candle, or burning coals, there is no evident reafon why it might not, in like manner, take fire from a fpontaneous flame, in contact with it over a great extent of furface.

Of the fifth remark, relative to the parts fpared by the fire, the author fuppofes the caufe to be in their lefs propenfity to burn.

In proof of the fixth, he cites a familiar example of water thrown on greafy and fpirituous fubftances, which does but augment the activity of the fire.

He attributes the little damage done to the combuftible clothing of the dead body to the nature of the flame, fimilar to that of the alcohol. Befide which, it is obferved, that the combuftion is performed almoft without flame, the body being flowly confumed like the pyrophore, and the nature of the afhes and greafe that remain, being the fame as thofe of all animal parts, the emanation of which has not been excited to the utmoft.*

Thefe explanations being given, the author yields to fuch reflections as refult from, and are the end of, his inquiries. The firft is, that accidental human combuftion might be fuppofed the work of the guilty; and the innocent might thus be involved in great misfortunes. He cites, in proof, the example of a man condemned to death, in confequence of fuch an accident. He thence very judicioufly obferves, that thefe facts

* Dont l'emanation n'a pas été portée au dérnier dégré.

facts are as neceſſary to be known by the lawyer as the natural hiſtorian.

He concludes with preſuming, that the terror which accidents ſo dreadful muſt inſpire, were they ſufficiently known, might perhaps deter many people from the vice of drunkenneſs.

However plauſible the reaſons given in this work may be of the propenſity of certain living animal bodies to catch fire, and be thus conſumed, it muſt be owned, that were they merely hypothetical, the poſſibility of an event ſo aſtoniſhing muſt be doubted; and that nothing leſs than experience could deſtroy the incredulity felt at the recital of theſe ſtrange combuſtions: but this authority of experience is ſo well ſupported by the number and agreement of the facts, and by the character of the perſons who teſtify their veracity, that none but the obſtinate can doubt of their exiſtence.

The work is written with perſpicuity and method, is rich in well-ſelected quotations, and the arguments it contains are always ſupported by facts, and ſtated with ſagacity.

